# miR‐31/QKI‐5 axis facilitates cell cycle progression of non‐small‐cell lung cancer cells by interacting and regulating p21 and CDK4/6 expressions

**DOI:** 10.1002/cam4.5309

**Published:** 2022-09-29

**Authors:** Wangyu Zhu, Yun Yu, Kexin Fang, Sisi Xiao, Lianli Ni, Changtian Yin, Xiangjie Huang, Xinchen Wang, Yongkui Zhang, Han‐Bo Le, Ri Cui

**Affiliations:** ^1^ Cellular and Molecular Biology Laboratory Affiliated Zhoushan Hospital of Wenzhou Medical University Zhoushan Zhejiang China; ^2^ Lung Cancer Research Center Zhoushan Hospital Zhoushan Zhejiang China; ^3^ Cancer and Anticancer Drug Research Center School of Pharmaceutical Sciences Wenzhou Medical University Wenzhou Zhejiang China; ^4^ Department of Cardio‐Thoracic Surgery Zhoushan Hospital Zhoushan Zhejiang China

**Keywords:** CDK4/6, cell cycle, MicroRNA‐31, non‐small‐cell lung cancer, P21, QKI‐5

## Abstract

**Background:**

RNA‐binding protein Quaking‐5 (QKI‐5), a major isoform of QKIs, inhibits tumor progression in non‐small cell lung cancer (NSCLC). However, the underlying molecular mechanisms of QKI‐5 in the cell cycle of NSCLC are still largely unknown.

**Methods:**

MTT, flow cytometry, and colony formation assays were used to investigate cellular phenotypic changes. Mice xenograft model was used to evaluate the antitumor activities of QKI‐5. Co‐immunoprecipitation, RNA immunoprecipitation (RIP), and RIP sequencing were used to investigate protein–protein interaction and protein–mRNA interaction.

**Results:**

The QKI‐5 expression was downregulated in NSCLC tissues compared with that in paired normal adjacent lung tissues. Overexpression of QKI‐5 inhibited NSCLC cell proliferative and colony forming ability. In addition, QKI‐5 induced cell cycle arrest at G0/G1 phase through upregulating p21^Waf1/Cip1^ (p21) expression and downregulating cyclin D1, cyclin‐dependent kinase 4 (CDK4), and CDK6 expressions. Further analyses showed that QKI‐5 interacts with p21 protein and CDK4, CDK6 mRNAs, suggesting a critical function of QKI‐5 in cell cycle regulation. In agreement with in vitro study, the mouse xenograft models validated tumor suppressive functions of QKI‐5 in vivo through altering cell cycle G1‐phase‐associated proteins. Moreover, we demonstrated that QKI‐5 is a direct target of miR‐31. The QKI‐5 expression was anticorrelated with the miR‐31 expression in NSCLC patient samples.

**Conclusion:**

Our results suggest that the miR‐31/QKI‐5/p21‐CDK4–CDK6 axis might have critical functions in the progression of NSCLC, and targeting this axis could serve as a potential therapeutic strategy for NSCLC.

## Graphical abstract

miR‐31 mediated QKI‐5 downregulation promoted tumor growth of NSCLC through regulating p21 protein and CDK4/6 mRNAs.

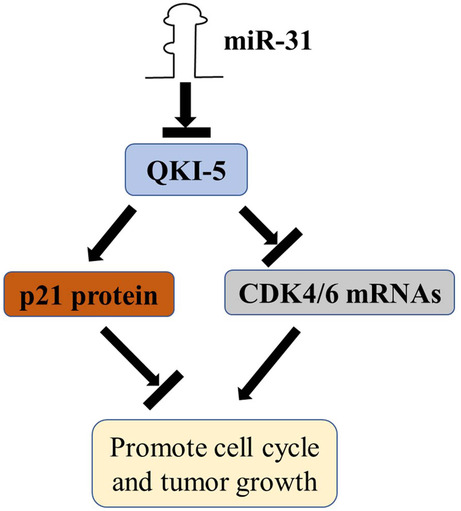

## 1 INTRODUCTION

Lung cancer is one of the great threats to human health due to its considerable cancer‐related mortality worldwide. Non‐small‐cell lung cancer (NSCLC) accounts for over 80% of lung cancer cases, and its main pathological subtypes include adenocarcinoma, squamous cell carcinoma, and large cell carcinoma.^1^, ^2^ NSCLC initiation and progression are related to a number of genetic and epigenetic changes, leading to the uncontrollable proliferation and aggressive metastasis.^3^ The SEER registry data for lung cancer patients who were diagnosed from 2009 through 2015 showed that the 5‐year relative survival rate was lower than 20%.^2^ Thus, further study needs to investigate underlying molecular mechanisms of NSCLC progression and metastasis to find novel therapeutic targets.

QKI is an RNA‐binding protein that belongs to the signal transduction and activation of the RNA (STAR) family. The QKI has four isoforms (QKI‐5, QKI‐6, QKI‐7, and QKI‐7b) based on its different C‐terminals.^4^ The QKI gene was broadly expressed in lung, prostate, colon, stomach, heart, testis, and myeloid lineage.^5^, ^6^ Accumulating evidence have suggested that QKI‐5 was a major isoform of QKIs in various cancers^7^ and inhibited cancer cell growth and metastasis.^8^, ^9^, ^10^, ^11^, ^12^ QKI‐5 could selectively bind to the QKI response element (QRE, 5′‐A[C/A]UAA‐3′) on cancer‐related genes, such as ß‐catenin.^8^ In addition, miR‐200‐ and miR‐375‐regulated epithelial–mesenchymal transition (EMT)‐associated alternative splicing changes by suppressing QKI‐5.^13^ miR‐155 directly targets QKI that results in the acquisition of cancer stem cell‐like (CSC‐like) properties in liver cancer.^14^ In addition, QKI‐5 inhibited lung cancer progression and metastasis by regulating circRNA, ß‐catenin signaling pathway, or TGF‐β/SMAD signaling.^11^, ^15^, ^16^, ^17^

MiR‐31 expression was upregulated in various cancers, including lung cancer.^18^, ^19^ Our previous study has shown that miR‐31 expression was increased in lung adenocarcinoma tissues with lymph node metastasis compared with those without lymph node metastases. In addition, miR‐31 facilitated cell migratory, invasive, and proliferative ability of NSCLC cells through activating the ERK1/2 signaling pathway.^20^ Furthermore, miR‐31 inhibited the negative regulator of RAS/MAPK, promoting the mutant KRAS‐mediated lung tumorigenesis.^19^ However, the association between miR‐31 and QKI‐5, and its function in the tumorigenesis of NSCLC are still undefined.

Our study demonstrated that QKI‐5 suppressed NSCLC cell proliferation both in vitro and in vivo by inhibiting cell cycle progression by regulating cell cycle G1 phase‐related proteins. Particularly, we demonstrated that QKI‐5 binds to the p21 protein, and CDK4/6 mRNAs that result in the alternation of these proteins’ expression. Furthermore, we found that miR‐31 directly targeted QKI‐5, and its expression was significantly anticorrelated with the *QKI‐5* expression in NSCLC tissues. Together, our study demonstrated that the miR‐31/QKI‐5/p21‐CDK4‐CDK6 pathway is closely involved in the progression of NSCLC.

## 2 MATERIALS AND METHODS

### 2.1 Patient samples

Fresh frozen NSCLC tissues and corresponding adjacent noncancer tissues were obtained from Zhoushan Hospital, Zhejiang Province, China between 2015 and 2016. All tissues were surgically resected and frozen in liquid nitrogen within 30 minutes and were stored at −80°C. Two board‐certified pathologists assessed the histological subtype by evaluating the hematoxylin and eosin‐stained slides in accordance with the World Health Organization (WHO) classification and the International Association for the Study of Lung Cancer, American Thoracic Society and European Respiratory Society (IASC/ATS/ERS).^21^ In addition, pathological tumor node metastasis (TNM) staging was evaluated based on the proposed 8th edition of lung cancer classification.^22^, ^23^, ^24^ Patients with NSCLC who received preoperative chemotherapy and radiotherapy were excluded. The clinicopathologic features of 30 patients with NSCLC were listed in Table 1. The correlation between QKI‐5 mRNA and miR‐31 was evaluated by using the TCGA NSCLC database. This study was approved by the Ethics Committee of Zhoushan Hospital with the patient^'^s written informed consent.

**TABLE 1:** Clinical characteristics of 30 non‐small‐cell lung cancer patients

Characteristics	Number
Age (mean), years	
≤ 60	10
> 60	20
Gender	
Male	16
Female	14
Tobacco smoking history	
Never	15
Current	15
Tumor location	
Upper right lobe	6
Lower right lobe	10
Upper left lobe	11
Lower left lobe	3
Tumor size, cm	
≤ 1.0	2
>1.0 ‐ ≤ 2.0	8
>2.0 ‐ ≤ 3.0	9
>3.0	11
Lymph node metastasis	
No	23
Yes	7
Histological subtype	
Adenocarcinoma	17
Squamous carcinoma	13
Pathological stage	
I A	15
I B	3
IIA	2
IIB	5
IIIA	3
IIIB	2

### 2.2 Cell lines and culture conditions

Human NSCLC cell lines (A549, HCC827, H1299, H1395, H1975, and H460), human bronchial epithelium (HBE) cell line, and 293 T cells were purchased from Cell Resource Center, Shanghai Institute of Life Sciences, Chinese Academy of Sciences. Lung cancer cells were cultured with RPMI‐1640 medium (Gibco, USA) containing 10% FBS (Gibco) and L‐glutamine. HEK293T cells were cultured in DMEM supplemented with 10% FBS. All cells were cultured in an incubator at 37°C with a humidified atmosphere of 5% CO_2_.

### 2.3 Quantitative reverse transcription‐PCR (qRT‐PCR)

Total RNAs were extracted from fresh tissues or cells using TRIzol reagent (Invitrogen, USA) and the concentration of mRNA was determined by Quawell 3000 UV Spectrophotometer (Quawell Technology, Inc). One microgram of total RNA was reverse transcripted using M‐MLV reverse transcriptase (Promega, USA), and qRT‐PCR was carried out using ABI 7500 Real‐Time PCR system (Applied Biosystems, USA) with SYBR Green reagents (Promega, USA). The specific primers were synthesized from Shanghai Generay Biotec Co, China. The primers used for the qRT‐PCR were QKI‐5, 5^'^‐CACCGCACCTAATACACC‐3^'^ (forward), 5^'^‐CCAAACGGAACTCCTCAC‐3^'^(reverse); GAPDH, 5^'^‐GACCTGACCTGCCGTCTA‐3^'^(forward), 5^'^‐AGGAGTGGGTGTCGCTGT‐3^'^(reverse). 2^−ΔΔCt^ method was used to evaluate the relative expression of mRNA.

### 2.4 Western blot analysis

Total proteins were isolated from fresh tissues and cells using lysis buffer with protease inhibitors. Cell lysates were subjected to SDS‐PAGE and transferred to the PVDF membrane. The membranes were incubated overnight at 4°C with specific antibodies: QKI‐5 (Millipore, 1:500), CDK4, CDK6, p18, p21 (Cell signaling, 1:1000), and GAPDH (Biosharp, 1:1000). After washing with TBST, The membranes were incubated with HRP‐conjugated secondary antibody at room temperature for 2 h. Signals were captured by Supersignal West Pico Chemiluminescent Substrate (Thermo Fisher Scientific, USA).

### 2.5 Lentiviral vector package and infection

GV358 and GV248 vectors were selected to establish QKI‐5 overexpressing or knockdown lentiviral vectors, respectively (EGFP expression). The sequencing verified vectors were transfected into HEK293T cells and the virus was collected after 48 h transfection. NSCLC cells were infected with lentivirus in RPMI‐1640 medium with 8 μg/ml polybrene. Five days later, the lentivirus‐infected cells were investigated for the expression of EGFP and the EGFP positive expression >90% was considered to be further used. QKI‐5 expression was determined by qRT‐PCR and Western blot analysis.

### 2.6 Cell proliferation and colony formation assays

Cell proliferation was assessed by a CCK‐8 cell‐counting kit (7 sea biotech, Shanghai, China). Cells were seeded in 96‐well plates at a density of 1 × 10^3^ cells/well. After 72 h incubation, 10 μl of CCK‐8 solution was added to each well and then incubated for 2 h at 37°C. The absorbance value was detected at 450 nm in triplicate from three independent experiments, and the growth curves were portrayed according to the optical data. For colony formation assay, 1000 (H1299/shCont and H1299/shQKI5) or 2000 (H460/Cont and H460/QKI5) cells in an RPMI‐1640 medium containing 10% FBS were seeded in a 6‐well plate and incubated at 37°C for 7–10 days. After fixation with 4% paraformaldehyde for 15 min, the cells were stained with crystal violet (Beyotime, China). ImageJ software was used to calculate colony numbers.

### 2.7 Cell cycle analysis

H1299 cells transfected with Lentiviral vector sh‐QKI‐5(LV‐sh‐QKI‐5) and H460 cells transfected with Lentiviral vector QKI‐5(LV‐QKI‐5) were harvested and fixed with fixation solution for 10 min, then permed in perm buffer II for 10 min, and stained in propidium iodide (PI) for 30 min at 4°C according to the manufacture protocols (BD, USA). About 20,000 cells per sample were collected and evaluated by flow cytometer (BD, USA).

### 2.8 Luciferase reporter assays

3'‐UTR of QKI‐5 were inserted into GV 306 plasmids and constructed by Shanghai Genechem Co., Ltd. The potential miR‐31 binding motif TCTTGCC was replaced with GAGGTAA. Then HEK 293 T cells (1 × 10^4^) were plated in a 24‐well plate and transfected with 0.2 μg of QKI‐5 3'‐UTR‐mutant or wildtype vector plus miR‐31 mimic (10 pmol) or control mimic (10 pmol) using Lipofectamine 3000 (Thermo Fisher Scientific). After 24 h transfection, luciferase activity was evaluated by Luciferase Assay Kit (Promega).

### 2.9 Co‐immunoprecipitation

The interactions of QKI‐5 and p21 proteints were detected by Co‐IP. RIPA lysis buffer (Beyond, China) was used to extract total proteins. The A/G agarose beads (Thermo Fisher, USA) were added to the protein solution and then incubated with anti‐QKI‐5 antibody overnight on a spinning wheel at 4°C. The antigen–antibody complexes were washed and suspended with protein lysate and proceeded to Western blot.

### 2.10 RNA immunoprecipitation, RIP sequencing, and qRT‐PCR

RNA immunoprecipitation (RIP) was conducted using H1299 lung cancer cells with 70–80% confluence. Cells were washed, harvested, and lysed with RIP lysis buffer containing protease inhibitors cocktail and RNase inhibitor and incubated at 4°C for 30 min. The lysates were centrifuged at 13000 × g for 20 min and collected supernatants. The anti‐QKI antibody/protein A/G beads or control IgG/protein A/G beads were added to the obtained supernatant and incubated overnight at 4°C with general rotation. Pellet beads and wash with RIP buffer. After treatment with proteinase K, immune complexes were heated at 55°C for 30 min to digest the protein. Immunoprecipitated RNAs were purified with phenol/chloroform extraction and subsequent ethanol precipitation. Purified RNAs were subjected to RNA‐seq and qRT‐PCR. RNA‐seq library was prepared with the TruSeq Stranded Total RNA Library Prep Kit (Illumina) and sequenced by the Illumina Hiseq2000 platform. The raw data were analyzed by the fragments per kilobase of transcript per million (FPKM) values across all the exons. For the RIP‐seq analysis, peak calling and peak annotation were analyzed using Homer. RIP results were then verified by real‐time PCR. The specific primers were CDK4 forward primer (5'‐GATGACTGGCCTCGAGATGT‐3^′^), CDK4 reverse primer (5'‐AGGCAGAGATTCGCTTGTGT‐3^′^), CDK6 forward primer (5'‐AGCAGGGGA‐ TTTTCATGTTG‐3^′^), and CDK6 reverse primer (5^′^‐ TGTGGCTCTATGTGTGCTGA‐3^′^).

### 2.11 Animal experiment

All animal experimental procedures were approved by the Animal Experimental Ethical Inspection of Laboratory Animal Center, Zhejiang Ocean University. BALB/c (nu/nu) nude mice were divided into LV‐QKI‐5 and LV‐control groups (n = 4 for each group). H460 cells (2 × 10^6^ cells) with stable overexpressed QKI‐5 or empty vector were injected into the flank of the nude mice subcutaneously. Caliper was used to measure tumor diameters every 2 days after tumors presented, and the volume was calculated according to the formula as follows: (Volume = length×width^2^×0.5236). The mice were euthanized, sacrificed, and photographed after 24 days of injection, and then xenograft tissues were removed and weighed.

### 2.12 Immunohistochemistry

The expression of QKI‐5, cyclin D1, Ki‐67, and CD31 in xenograft tissues were evaluated by using immunohistochemistry (IHC) staining. The tumor tissues were fixed with 10% neutralized formalin and the paraffin‐embedded tissue samples were sliced into 4‐μm sections. After deparaffinized, the slides were boiled with 10 mM citrate buffer and blocked with 10% goat serum. Then the slides were incubated with the anti‐QKI‐5 (Millipore, 1:300), anti‐cyclin D1 (cell signaling, 1:100), anti‐Ki‐67 (Abcam, 1:200), and anti‐CD31 (cell signaling, 1:100) at 4°C overnight. The slides were further incubated with the secondary antibody with HRP and visualized by DAB.

### 2.13 Statistical analysis

All statistical analyses were performed using SPSS 17.0 and GraphPad Prism 5.0 software. The measurement data of the experiments were expressed as mean values with standard deviations and evaluated using the Student's *t* test. The data of QKI‐5 mRNA and miR‐31 were obtained from the TCGA database (https://tcga‐data.nci.nih.gov/tcga). Kaplan–Meier survival plots of QKI‐5 mRNA were portrayed using an online survival analysis tool (http://www.kmplot.com). The statistical significance was defined as *p* < 0.05.

## 3 RESULTS

### 3.1 QKI‐5 expression is downregulated in NSCLC tissues

**FIGURE 1:**
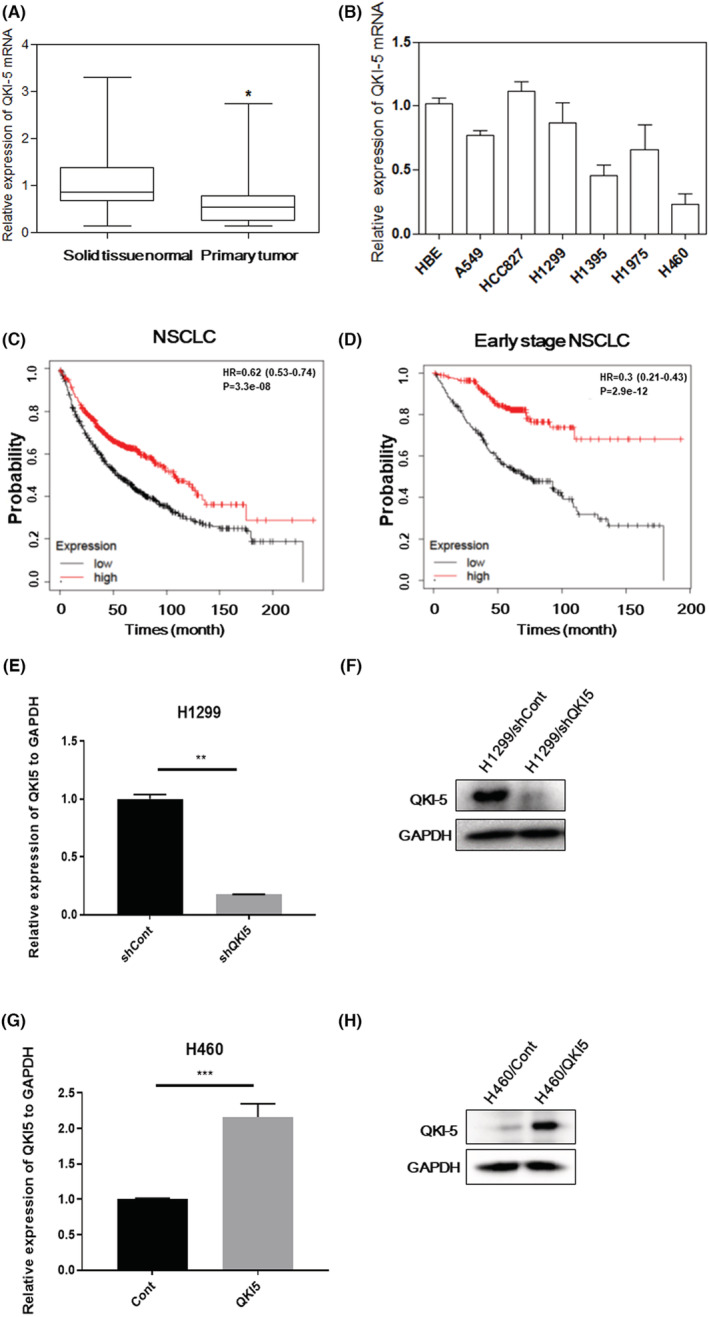
QKI‐5 is downregulated in non‐small‐cell lung cancer (NSCLC). (A) qRT‐PCR analysis of QKI‐5 mRNA levels in 31 NSCLC tissues and corresponding normal adjacent tissues (*p* = 0.0277). GAPDH was used to normalize QKI‐5 expression levels. (B) qRT‐PCR analysis of QKI‐5 mRNA levels in various lung cancer cell lines. GAPDH was used to normalize QKI‐5 expression levels. Kaplan–Meier survival curves for 1882 patients with NSCLC (C) (*p* < 0.0001) and 774 patients with stage I NSCLC (D) (*p* < 0.0001). qRT‐PCR (E) and Western‐blot analysis (F) measures QKI‐5 mRNA and protein expressions in H1299 cells transfected with shQKI‐5 plasmid. qRT‐PCR (G) and Western‐blot (H) analysis measures QKI‐5 mRNA and protein expressionls in H460 cells transfected with QKI‐5 overexpressing plasmid (*p < 0.05, ** *p* < 0.01, *** *p* < 0.001).

The QKI‐5 mRNA expression was measured by qRT‐PCR in 30 matched primary NSCLC samples as well as their corresponding noncancerous tissue samples. The QKI‐5 mRNA expression was markedly downregulated in primary NSCLC tissue samples compared with those in normal lung tissue samples (Figure 1A). In addition, the QKI‐5 mRNA expression was reduced in several human NSCLC cell lines compared with that in the human bronchial epithelial cell line (Figure 1B). Kaplan–Meier survival analysis using 1882 patients with NSCLC (http://www.kmplot.com) revealed that the low QKI mRNA expression was associated with short overall survival (OS) (*p* < 0.0001, Figure 1C). The low and high expression groups were separated according to the median value of QKI mRNA expression. Of note, the median survival of the low expression group is 54.47 months and the high expression group is 107 months. Particularly, the decreased QKI mRNA expression was significantly associated with worse overall survival in 774 stage I NSCLC patients (*p* < 0.0001, Figure 1D).

Our previous study demonstrated that H1299 cells have relatively high expression of QKI‐5, whereas H460 cells have relatively low expression of QKI‐5.^25^ To explore the biological functions of QKI‐5 in NSCLC, we transfected QKI‐5 overexpressing vector, LV‐QKI‐5 into the H460 cells or QKI‐5 knockdown vector, LV‐sh‐QKI‐5 into the H1299 cells. Lentiviral vector encoding nonsense sequence was served as control. Knockdown of QKI‐5 with LV‐sh‐QKI‐5 in H1299 cells resulted in downregulated expressions of QKI‐5 mRNA and protein (Figure 1E,F). Conversely, transfection of LV‐QKI‐5 significantly increased the QKI‐5 mRNA and protein expressions in H460 cells (Figure 1G,H).

### 3.2 QKI‐5 inhibited cell proliferation and cell cycle progression in NSCLC cells

**FIGURE 2:**
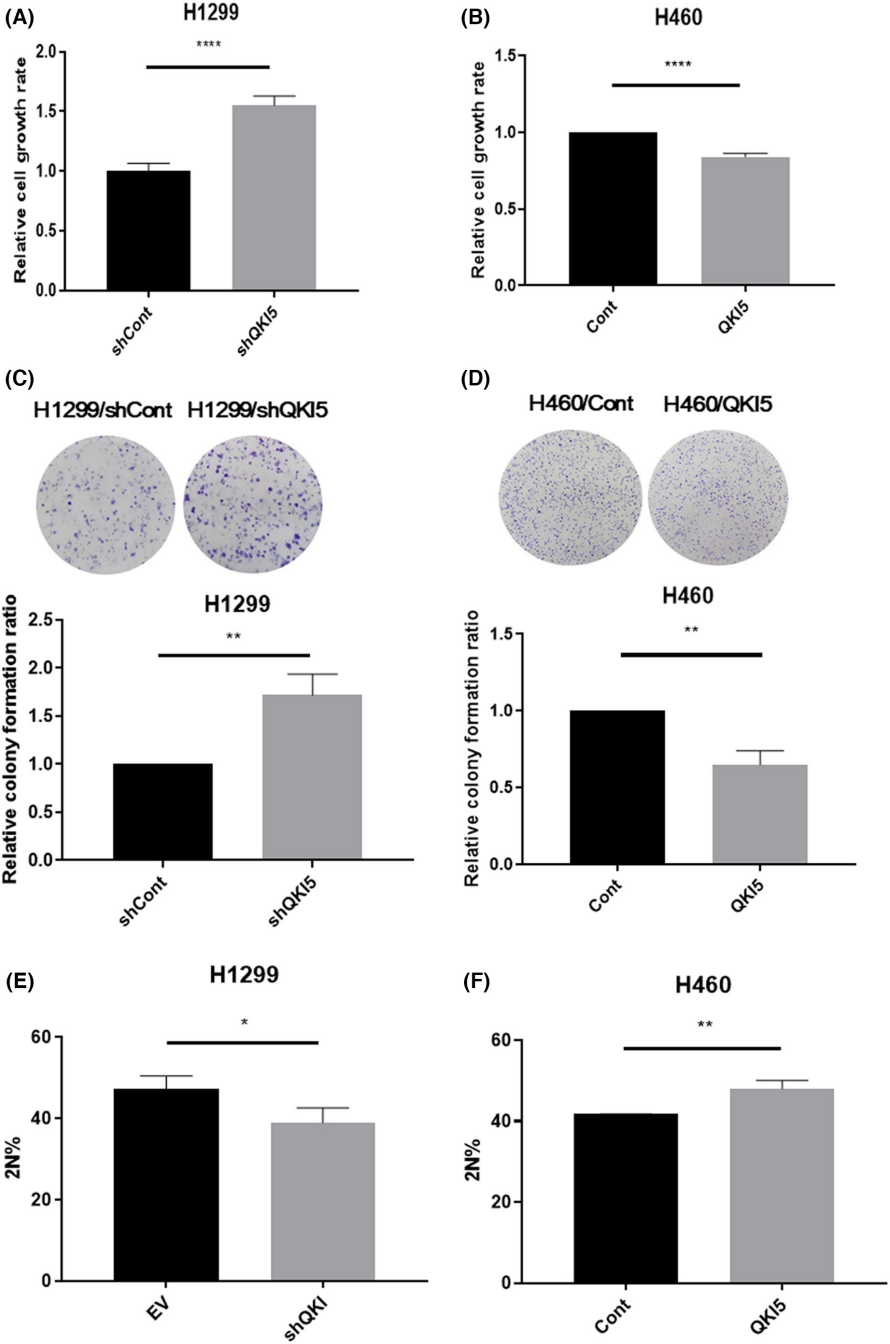
QKI‐5 inhibits tumor progression in non‐small‐cell lung cancer. CCK‐8 assays in QKI‐5 knockdown H1299 cells (A) and QKI‐5 overexpressing H460 cells (B). Colony forming assay in QKI‐5 knockdown H1299 cells (C) and QKI‐5 overexpressing H460 cells (D); Cell cycle analysis was conducted by flow cytometry in QKI‐5 knockdown H1299 cells (E) and QKI‐5 overexpressing H460 cells (F). Percentage of cells in G0/G1 phase was calculated (*p < 0.05, ** *p* < 0.01, ***p < 0.001).

CCK‐8 assays and colony forming assays showed that knocking down QKI‐5 in H1299 cells promoted cell growth and colony forming ability, whereas overexpression of QKI‐5 inhibited H460 cells proliferative and colony forming ability (Figure 2A–D). We further investigated whether QKI‐5 could affect cell proliferation by inhibiting cell cycle progression in NSCLC cells. Cell cycle analysis indicated that the knockdown of QKI‐5 in H1299 cells reduced the percentage of G0/G1 phase cells, whilst QKI‐5 overexpression in H460 cells increased the percentage of G0/G1 phase cells (Figure 2E,F).

### 3.3 QKI‐5 inhibited cell cycle progression by altering G1‐phase‐associated protein expressions

**FIGURE 3:**
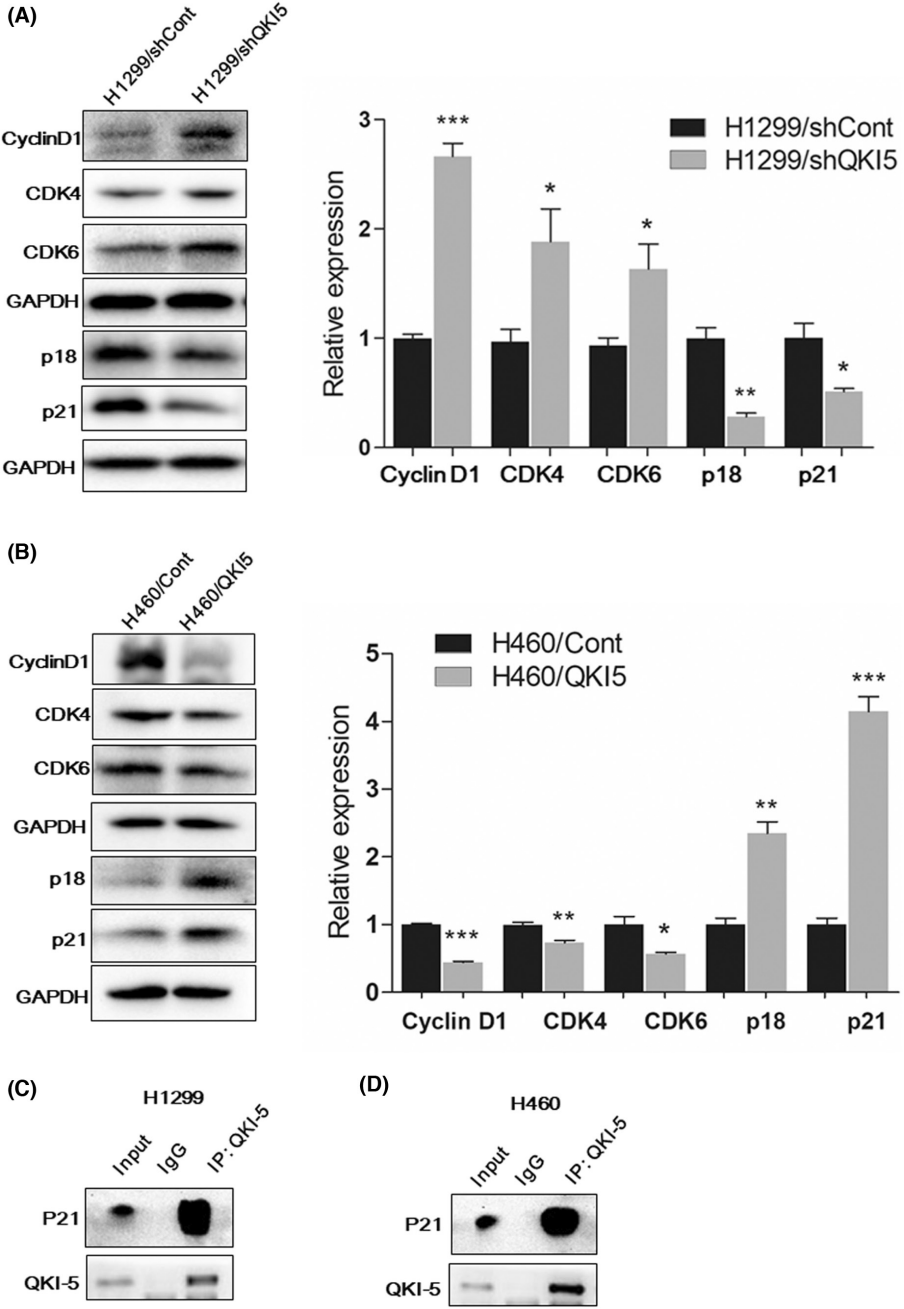
QKI‐5 altered the expression of cell cycle G1‐phase‐associated proteins. (A) Expression of cell cycle G1‐phase‐associated proteins (p18, p21, cyclin D1, CDK4, and CDK6) in QKI‐5 knockdown H1299 cells and control cells were measured by western blot analysis. (B) Expression of cell cycle G1‐phase‐associated proteins (p18, p21, cyclin D1, CDK4, and CDK6) in QKI‐5 overexpressing H460 cells and control cells were measured by western‐blot analysis. The related bands were quantified by Image Lab 6.0.1 software. Co‐IP assay was used to verify the potential interaction of QKI‐5 and p21 in H1299 (C) and H460 cell lines (D). Input fractions and IgG were served as controls (* *p* < 0.05, ** *p* < 0.01, *** *p* < 0.001)

We further conducted western blot analysis to evaluate the cell cycle G1‐phase associated proteins after overexpression or knockdown of QKI‐5. Knocking down QKI‐5 in H1299 cells significantly elevated the expressions of Cyclin D1, CDK4, and CDK6 while reducing the expressions of p18 and p21 (Figure 3A). Conversely, overexpression of QKI‐5 in H460 cells markedly decreased Cyclin D1, CDK4, and CDK6 expression, whilst increased p18 and p21 expressions (Figure 3B). These results indicate that the growth inhibitory effect of QKI‐5 might be partly due to the G1‐phase cell cycle arrest in NSCLC. To investigate whether QKI‐5 interacts with these cell cycle‐related proteins and subsequently alters their expression levels, Co‐IP assay was conducted. Our results showed that QKI‐5 interacts with p21 but not with other cell cycle‐related proteins in both H1299 and H460 cells, suggesting the direct regulatory function of QKI‐5 to the p21 protein in NSCLC (Figure 3C,D).

**FIGURE 4:**
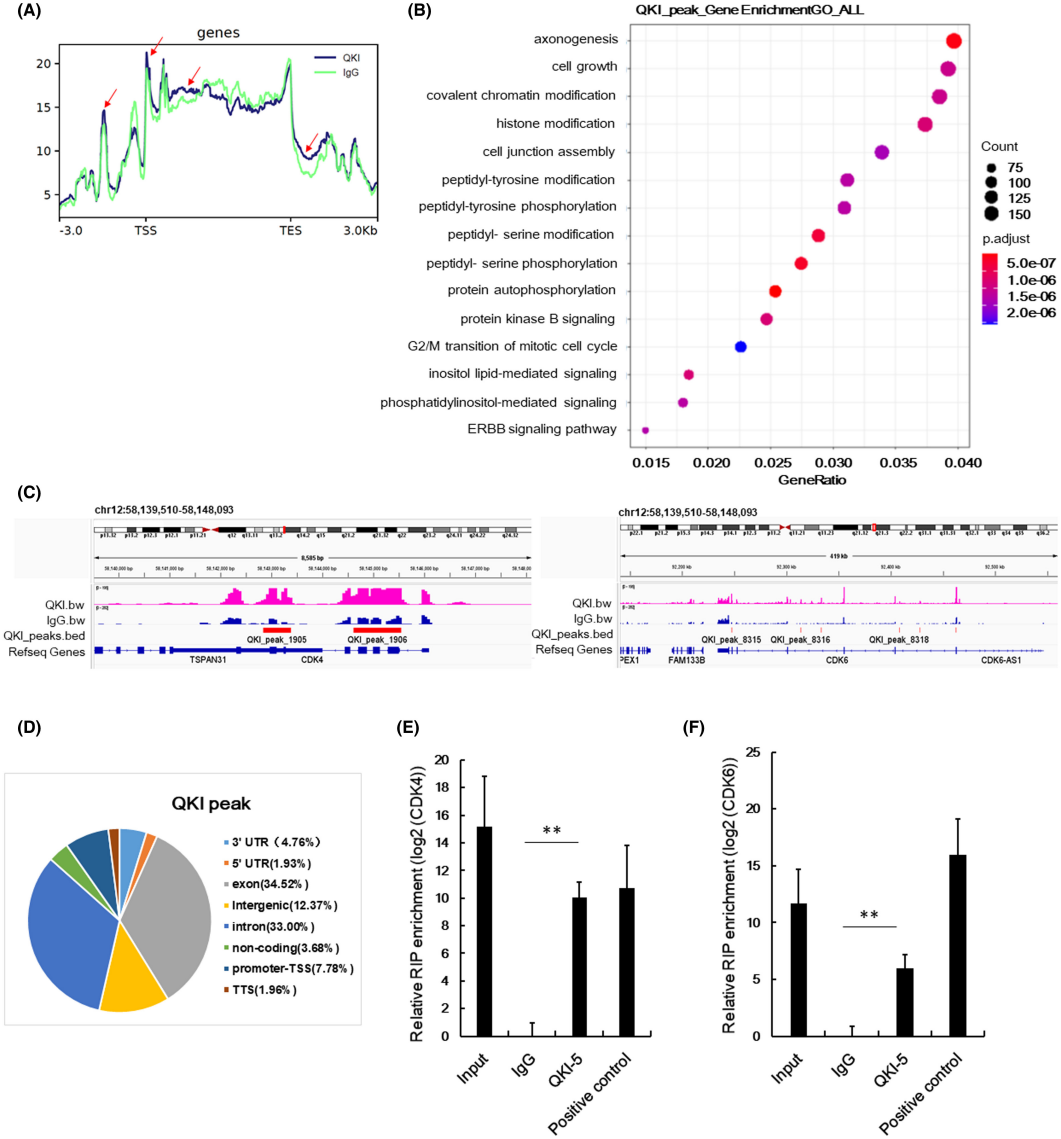
RIP‐seq and qRT‐PCR analysis identify direct mRNA targets of QKI‐5. (A) Lysate from H1299 cells was incubated with IgG and anti‐QKI‐5 antibody. Pull‐down RNA were assessed with RNA‐seq. The QKI peak read in the 3 KB upstream of transcription start site (TSS), gene body, and 3 KB downstream of transcription end sites (TES) was shown. Red arrow indicates different QKI peak reads between QKI group and IgG group. (B) The annotations of QKI‐binding mRNA were analyzed by gene ontology (GO) analysis. (C) Integrative Genomics Viewer (IGV) analysis was used to demonstrate the peaks of QKI‐5 and IgG. Magenta represents QKI peak, blue represents IgG peak, and red represents the different QKI peak reads between QKI and IgG groups. (D) The distribution of QKI peaks with functional regions was shown. The pull‐down RNA was evaluated by qRT‐PCR using specific primers for CDK4 (E) and CDK6 (F). Data are presented as mean ± SD as determined by triple assays (* *p* < 0.05, ** *p* < 0.01, *** *p* < 0.001)

Considering the critical functions of QKI‐5 on RNA binding, we also conducted RIP‐seq to identify direct mRNA targets of QKI‐5. The QKI peaks were demonstrated by comparing QKI‐5 overexpressing samples with the IgG samples. The QKI peak read in the 3 KB upstream of transcription start site (TSS), gene body, and 3 KB downstream of transcription end sites (TES) from RIP‐Seq analysis were shown in Figure 4A. As indicated in the red arrow, markedly different QKI peak reads were observed in the QKI group compared with the IgG group. Subsequent gene ontology (GO) analysis indicated that some pathways were associated with QKI peaks including cell growth and G2/M transition of mitotic cell cycle pathways (Figure 4B). Both CDK4 and CDK6 peak read in an integrative genomic viewer indicated that QKI peaks 1905 and 1906 in CDK4, and QKI peaks 8315, 8316, and 8318 in CDK6 were higher in the QKI group than the IgG group (Figure 4C). The functional regions of QKI peaks were distributed in exon, intron, 5^,^‐UTR, 3^,^‐UTR, promoter, distal intergenic, and noncoding RNA (Figure 4D). Further analysis of RIP‐seq data showed that QKI‐5 could bind to CDK4 exon and CDK6 5^’^‐UTR (Table 2). RIP qRT‐PCR analysis further validated that CDK4 and CDK6 mRNAs were highly expressed in RIP samples using an antibody against QKI‐5 compared with the RIP samples using immunoglobulin G (IgG) (Figure 4E,F). These data suggest that QKI‐5 inhibits CDK4 and CDK6 protein levels partly through interacting with CDK4 and CDK6 mRNAs.

**TABLE 2:** The annotation of QKI peaks connected to CDK4 and CDK6 mRNA

Peak ID	Chr	Start	End	Peak Score	Annotation	Gene Name	Gene Description
QKI_peak_1906	chr12	58,144,612	58,145,548	13.56924	exon (NM_000075, exon 3 of 8)	CDK4	cyclin‐dependent kinase 4
QKI_peak_8319	chr7	92,427,890	92,428,223	9.84865	intron (NM_001145306, intron 2 of 7)	CDK6	cyclin‐dependent kinase 6
QKI_peak_8320	chr7	92,462,432	92,462,951	9.71974	5' UTR (NM_001259, exon 2 of 8)	CDK6	cyclin‐dependent kinase 6
QKI_peak_8318	chr7	92,408,256	92,408,455	7.31521	intron (NM_001145306, intron 2 of 7)	CDK6	cyclin‐dependent kinase 6

### 3.4 QKI‐5 inhibits tumor growth in vivo

**FIGURE 5:**
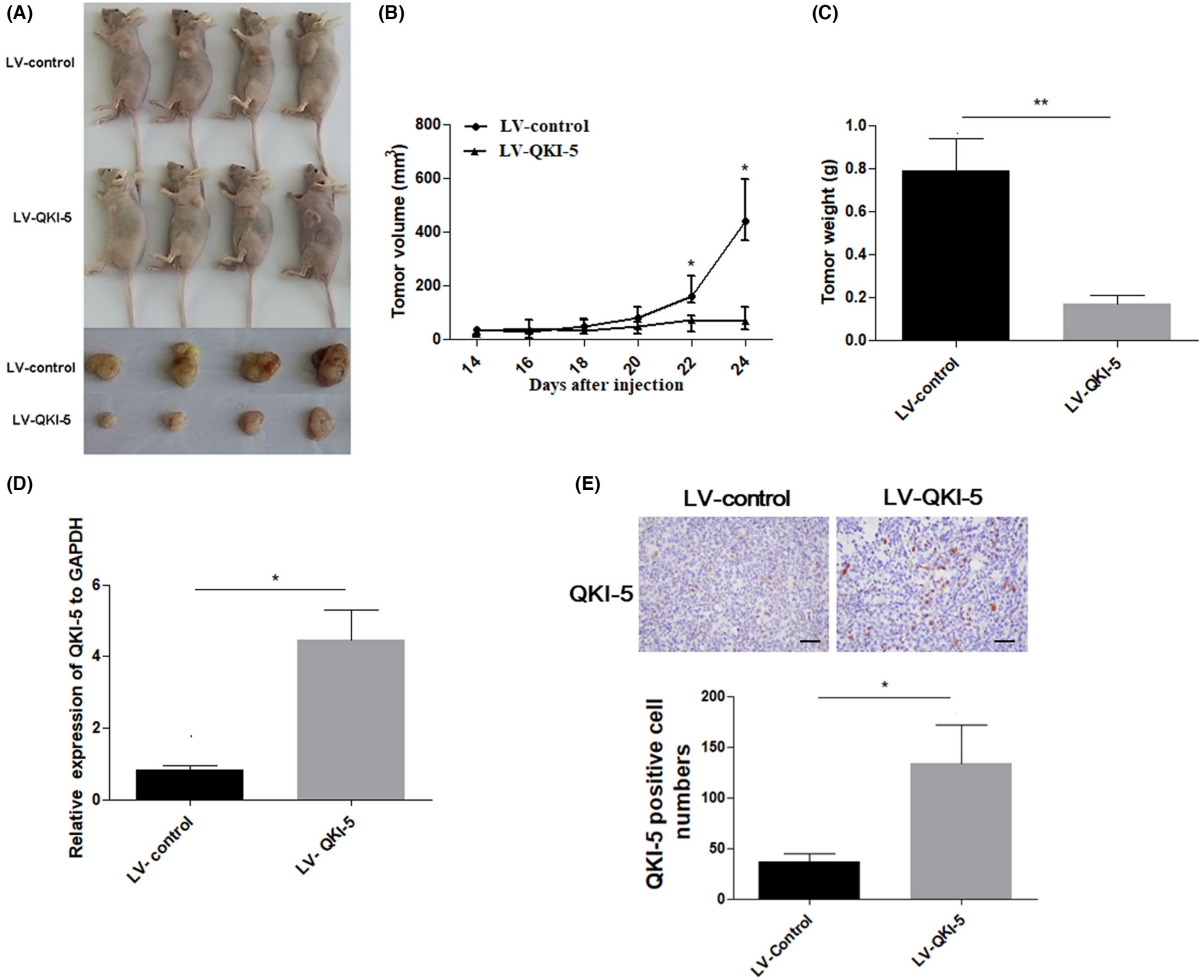
Overexpression of QKI‐5 inhibited tumor growth in xenograft mice model. (A) Representative images of H460 cell injected xenograft nude mice (up) and tumors (down) at the last day of the experiment. (B) Tumor volumes were measured every 2 days after 14 days inoculation with either the QKI‐5 overexpressing or control cells. (C) Tumor weights from mouse xenograft model injected with QKI‐5 overexpressing or control cells were measured at the end of the experiment (*n* = 4). (D) QKI‐5 mRNA expression in mice tumor tissues from QKI‐5 overexpressing H460 cells and control cells was analyzed by qRT‐PCR. (E) Immunohistochemistry analysis detects the number of QKI‐5 positive cells in eight fields of tumor sections that display highest reactivity with an antibody were counted at ×200 and presented as mean ± S.D (*p < 0.05, ** *p* < 0.01)

**FIGURE 6:**
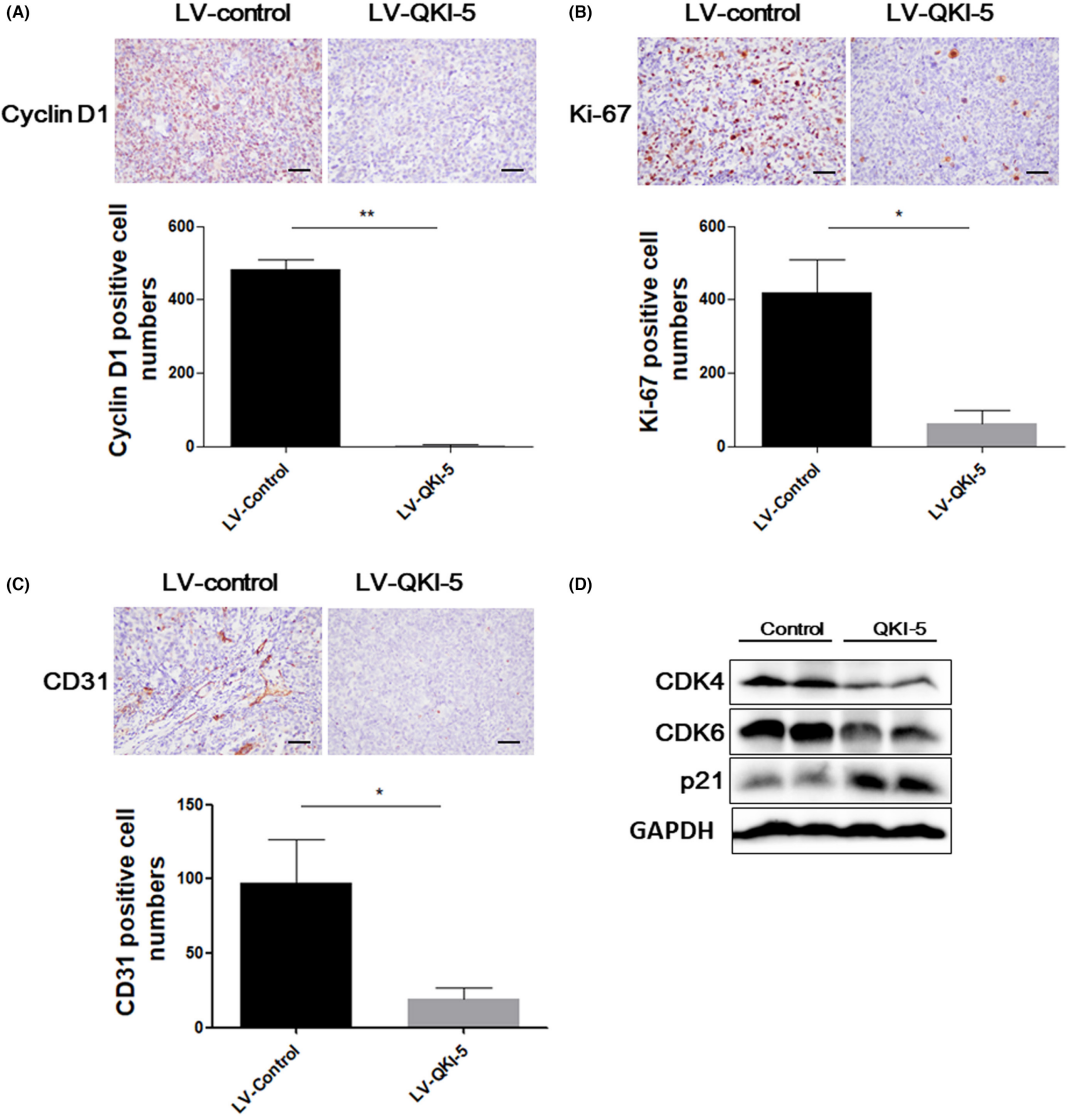
Cyclin D1, Ki‐67, and CD31 expressions were reduced in QKI‐5 overexpressing mice tumor tissues. Immunohistochemistry analysis detects cyclin D1, Ki67, and CD31 positive cells in tumors tissues derived from QKI‐5 overexpressing H460 cells or control cells. The number of cyclin D1 (A), ki‐67 (B) or CD 31 (C) positive cells in eight fields of tumor sections that display highest reactivity with antibodies were counted at ×200, respectively, and presented as mean ± S.D. (D) Western‐blot analysis detects CDK4, CDK6, and p21 protein expressions in xenograft tumors injected with QKI‐5 overexpressing H460 cells and control cells (*p < 0.05, ** *p* < 0.01)

To further assess the tumor suppressive effects of QKI‐5 in vivo, 2 × 10^6^ QKI‐5 overexpressing or control H460 cells were subcutaneously injected into nude mice. The tumor volumes were measured every 2 days after 14 days of injection. Tumors were resected and weighted on the last day of the experiments. We found that overexpressing QKI‐5 significantly suppressed tumor growth in vivo. Accordingly, the tumor volume and weight were markedly reduced in mice bearing QKI‐5 overexpressing H460 cells compared with those in mice bearing control cells (Figure 5A–C). qRT‐PCR and IHC analyses validated that QKI‐5 mRNA and protein levels were increased in tumor tissues from QKI‐5 overexpressing H460 cells injected mice (Figure 5D,E). IHC analyses also demonstrated that the Cyclin D1, Ki‐67, and CD31 expressions were significantly reduced in QKI‐5 overexpressing tumor tissues (Figure 6A‐C). Moreover, Western blot analysis showed that the CDK4 and CDK6 expressions were downregulated, whereas p21 expression was upregulated in tumor tissues from mice bearing QKI‐5 overexpressing cells compared with mice bearing control cells (Figure 6D). These findings indicated that QKI‐5 inhibited tumor growth in vivo by regulating cell cycle‐related proteins.

### 3.5 miR‐31 targets QKI‐5 in NSCLC

**FIGURE 7:**
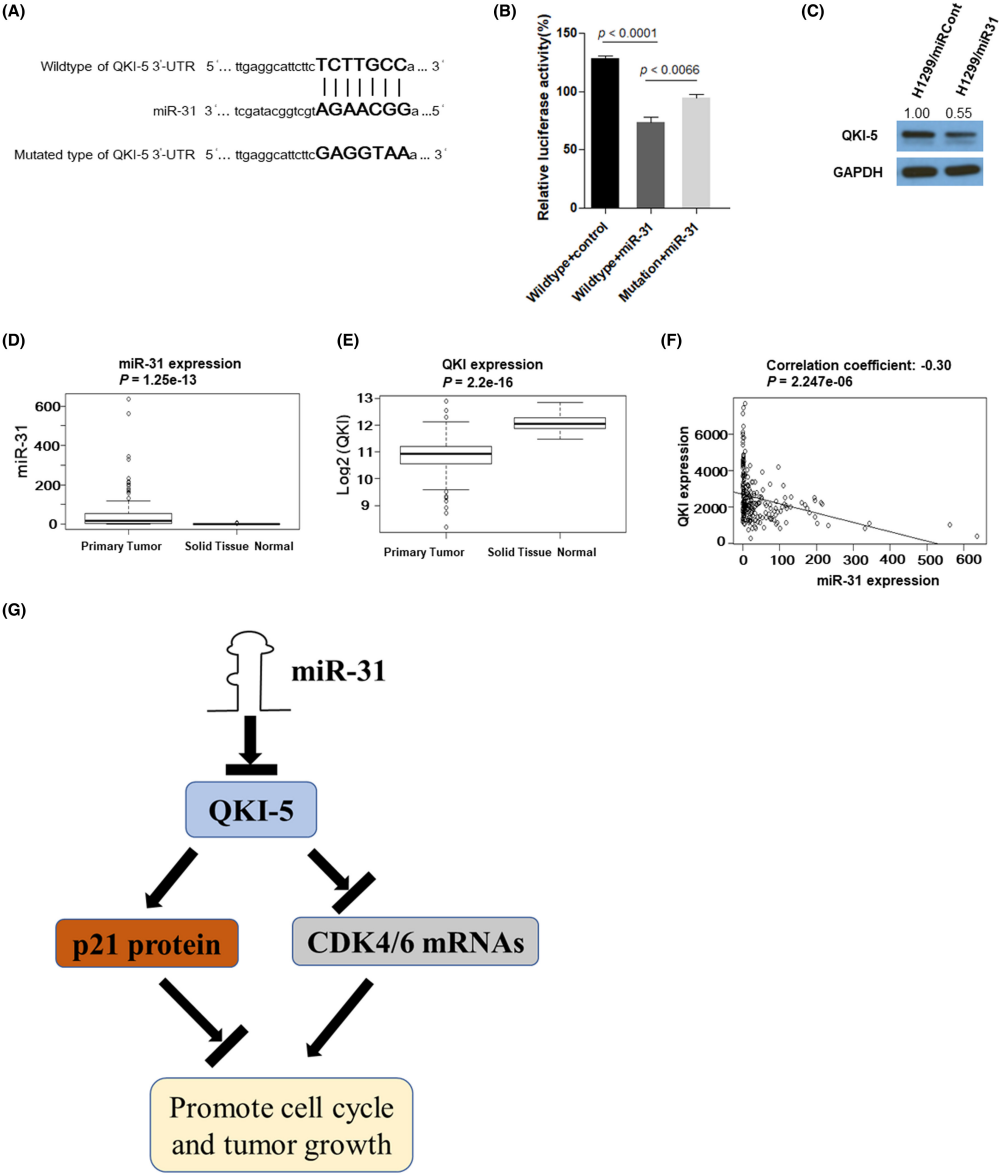
miR‐31 directly targets QKI‐5 in non‐small cell lung cancer (NSCLC). (A) miR‐31‐binding sites on 3'‐UTR of QKI‐5 were shown. (B) miR‐31 mimic was cotransfected with wild‐type or mutated form of QKI‐5 3'UTRs in luciferase reporter constructs into 293 T cells. Data are presented as mean ± SD and evaluated by triple assays. (C) Western blot analysis detects QKI‐5 expression in miR‐31 overexpressing H1299 cells and control cells. ImageJ software was used to quantify the band intensity and relative values were calculated by normalizing to the value of each corresponding GAPDH. (D) miR‐31 expression data were acquired from TCGA miR‐seq data set. (E) QKI expression data were acquired from TCGA RNA‐seq data set. (F) Correlation between miR‐31 and QKI in NSCLC was evaluated by using TCGA NSCLC data set (*n* = 306). (G) Proposed miR‐31‐mediated QKI‐5 downregulation promotes tumor growth of NSCLC by regulating p21 protein and CDK4/6 mRNAs (*p < 0.05, **p < 0.01)

Our previous research demonstrated that miR‐31 is an onco‐miRNA in NSCLC.^20^ We analyzed the 3'UTR of QKI‐5 by TargetScan (http://www.targetscan.org) and found that it has an miR‐31‐binding site (Figure 7A), suggesting QKI‐5 might be a target of miR‐31. Thus, we constructed the wild‐ and mutated‐type QKI‐5 3'‐UTR and conducted the luciferase reporter assays. HEK293T cells were transfected with 5 nM miR‐31 mimics and wild‐type QKI‐5 3'‐UTR reporter, mutated‐type QKI‐5 3'‐UTR reporter, or empty vector. Cotransfection of miR‐31 mimics with wild‐type QKI‐5 3'UTR significantly reduced luciferase activity. However, co‐transfection of miR‐31 mimics with mutated type QKI‐5 3'UTR significantly ameliorated the reduction of luciferase activity on wild‐type QKI‐5 3'UTR (Figure 7B). Moreover, transfection of miR‐31 mimics to H1299 cells reduced QKI‐5 protein expression (Figure 7C). To explore the correlation of QKI‐5 and miR‐31 in clinical specimens, we analyzed the data from the TCGA database. We found that the miR‐31 expression was significantly increased (Figure 7D), whereas QKI expression was reduced in primary lung tumor tissues compared with that in normal lung tissues (Figure 7E). Pearson correlation analysis showed that the miR‐31 expression was significantly anticorrelated with QKI‐5 in NSCLC patient samples (correlation coefficient = −0.30, *p* < 0.0001, Figure 7F). These results suggested that the reduced QKI‐5 expression is partly due to the enhanced expression of miR‐31 in NSCLC samples.

## 4 DISCUSSION

QKI family plays tumor suppressive functions in a number of cancers, including lung cancer.^6^, ^26^ QKI‐5 was a major form of QKIs and predominantly expressed in NSCLC. QKI‐5 negatively regulated miR‐196b‐5p in NSCLC and upregulated miR‐196b‐5p promoted cell migratory and proliferative ability by inhibiting tumor suppressors, TSPAN12 and GATA6.^25^ Several studies have reported that QKI inhibited cancer cell proliferation through cell cycle arrest by reducing cyclin D1 and cyclin E, and enhancing the stability of p27 mRNA.^9^, ^10^, ^27^, ^28^ Here, we further investigated the underlying molecular mechanism of QKI‐5 in NSCLC. QKI‐5 induced cell cycle arrest and inhibited the proliferation of NSCLC cells by interacting with p21 protein and CDK4/CDK6 mRNAs. QKI‐5 mediated alteration of these proteins subsequently inhibited cell cycle G1 to S transition. Moreover, we found that miR‐31 directly targets QKI‐5 in NSCLC cells.

To our knowledge, this study demonstrated for the first time that the cell cycle‐related proteins, p21, CDK4, and CDK6 were regulated by QKI‐5 in NSCLC cells. Immunoprecipitation analysis showed that QKI‐5 directly interacts with p21, suggesting that QKI‐5 might bind to and stabilize p21 protein, leading to an accumulation of p21 protein in NSCLC cells. In addition, the RIP‐seq and RIP‐qRT‐PCR analyses demonstrated that QKI‐5 could bind to CDK4 and CDK6 mRNAs. We speculate that QKI‐5 might influence the translation process of CDK4 and CDK6 mRNAs, which results in decreased protein expressions of CDK4 and CDK6 in NSCLC cells. CDK4/6 inhibitor has been demonstrated to prevent lung cancer progression with or without KRAS mutation.^29^, ^30^ Our study partly illustrated the potential mechanism of CDK4/6 inhibition by QKI‐5, and supported the theory that inhibiting CDK4/6 might be a potential therapeutic method for lung cancer. Further study needs to validate our hypothesis.

miRNAs work as post‐transcriptional regulators in tumorigenesis, and several studies showed that QKI is a target of miRNAs. Wu et al. revealed that miR‐9‐5p could directly target QKI‐5 in prostate cancer that results in promoting tumor progression.^31^ Moreover, the critical gatekeeper of the epithelial state, miR‐200c, could induce mRNA alternative splicing through suppressing QKI expression and impact cancer‐associated epithelial cell plasticity.^13^ In addition, miR‐143‐3p exerts tumor suppressive function in esophageal squamous cell carcinoma by directly targeting QKI‐5.^32^ Based on the above literature, we speculate that down‐regulated QKI‐5 expression in NSCLC might be related to dysregulated miRNAs expression. Our previous study has shown that miR‐31 was upregulated in NSCLC tissue samples and associated with lymph node metastasis.^20^ Thus we wondered whether miR‐31 could finely control QKI‐5 expression in NSCLC. Luciferase reporter and western‐blot assays demonstrated that QKI‐5 is a direct target of miR‐31. In agreement with our in vitro results, clinical tissue samples showed that miR‐31 was upregulated whereas QKI mRNA was downregulated in NSCLC, with a significant negative correlation between each other. These results indicate that miR‐31 mediated downregulation of QKI‐5 at least partly contributes to the accelerated NSCLC cell proliferation.

## 5 CONCLUSION

The present study demonstrated that the QKI‐5 might be a potential prognostic biomarker for NSCLC. QKI‐5 inhibited NSCLC cell growth by interacting with cell cycle related protein p21, and CDK4/6 mRNAs. In addition, we found that QKI‐5 is a target of miR‐31. Our study found that the miR‐31/QKI‐5/p21‐CDK4‐CDK6 axis might exert important functions in the progression of NSCLC, and targeting the miR‐31/QKI‐5/p21‐CDK4‐CDK6 axis could be an effective therapeutic method for NSCLC.

## ETHICS APPROVAL AND CONSENT TO PARTICIPATE

This study was conducted according to the guidelines of the Declaration of Helsinki and approved by the Ethics Committee of Zhoushan Hospital, Zhejiang, China (No.115, ethical review 2018, 3 August, 2018). Animal ethics approval (2020021) was received from Zhejiang Ocean University for Medical Research Animal Ethics Committee.

## AUTHOR CONTRIBUTIONS

**Wangyu Zhu:** Formal analysis (equal); funding acquisition (equal); investigation (equal); resources (equal); writing – original draft (equal). **Yun Yu:** Formal analysis (equal); investigation (equal); validation (equal); visualization (equal). **Kexin Fang:** Formal analysis (equal); funding acquisition (equal); methodology (equal). **Sisi Xiao:** Formal analysis (supporting); investigation (supporting); validation (supporting). **Lianli Ni:** Formal analysis (supporting); investigation (supporting). **Changtian Yin:** Formal analysis (supporting); validation (supporting). **Xiangjie Huang:** Formal analysis (supporting); visualization (supporting). **Xinchen Wang:** Investigation (supporting); validation (supporting). **Yongkui Zhang:** Resources (supporting); writing – review and editing (supporting). **Hanbo Le:** Resources (equal); writing – review and editing (equal). **Ri Cui:** Conceptualization (lead); resources (equal); supervision (lead); writing – original draft (equal); writing – review and editing (equal).

## FUNDING INFORMATION

This study was supported by grants from the Natural Science Foundation of Zhejiang Province (LQ17H160001 and LZ22H160006), Health Commission of Zhejiang Province (2020RC136 and 2022RC292), Health Commission of Zhoushan City (2018G01), Science and Technology Program of Zhejiang Province (GC20H160001), and National Natural Science Foundation of China (81672305).

## CONFLICT OF INTEREST

The authors declare that they have no conflict of interest.

## Data Availability

The data sets used and/or analyzed in this study are available from the corresponding author upon reasonable request.
